# The Stack: A New Bacterial Structure Analyzed in the Antarctic Bacterium *Pseudomonas* *deceptionensis* M1^T^ by Transmission Electron Microscopy and Tomography

**DOI:** 10.1371/journal.pone.0073297

**Published:** 2013-09-09

**Authors:** Lidia Delgado, Ornella Carrión, Gema Martínez, Carmen López-Iglesias, Elena Mercadé

**Affiliations:** 1 Crio-Microscòpia Electrònica. Centres Científics i Tecnològics, Universitat de Barcelona, Barcelona, Spain; 2 Laboratori de Microbiologia, Facultat de Farmàcia, Universitat de Barcelona, Barcelona, Spain; Miami University, United States of America

## Abstract

In recent years, improvements in transmission electron microscopy (TEM) techniques and the use of tomography have provided a more accurate view of the complexity of the ultrastructure of prokaryotic cells. Cryoimmobilization of specimens by rapid cooling followed by freeze substitution (FS) and sectioning, freeze fracture (FF) and observation of replica, or cryoelectron microscopy of vitreous sections (CEMOVIS) now allow visualization of biological samples close to their native state, enabling us to refine our knowledge of already known bacterial structures and to discover new ones.

Application of these techniques to the new Antarctic cold-adapted bacterium *Pseudomonas* *deceptionensis* M1^T^ has demonstrated the existence of a previously undescribed cytoplasmic structure that does not correspond to known bacterial inclusion bodies or membranous formations. This structure, which we term a “stack”, was mainly visualized in slow growing cultures of *P* *. deceptionensis* M1^T^ and can be described as a set of stacked membranous discs usually arranged perpendicularly to the cell membrane, but not continuous with it, and found in variable number in different locations within the cell. Regardless of their position, stacks were mostly observed very close to DNA fibers. Stacks are not exclusive to *P* *. deceptionensis* M1^T^ and were also visualized in slow-growing cultures of other bacteria. This new structure deserves further study using cryoelectron tomography to refine its configuration and to establish whether its function could be related to chromosome dynamics.

## Introduction

For many years, bacterial cytoplasm was thought to be a homogeneous compartment containing macromolecules and few structures of interest in comparison with eukaryotic cells. In most prokaryotes, when the cytoplasm is visualized by conventional transmission electron microscopy (TEM), it is only possible to observe irregular areas with a fibrous appearance corresponding to the nucleoid and a large amount of small granules scattered throughout the rest of the cytoplasm that correspond to ribosomes. In some prokaryotes, inclusions and vesicles involved in several physiological processes are also observed. To date, the main cytoplasmic structures described are: a) gas vesicles composed entirely of a protein shell characteristic of aquatic photosynthetic bacteria [1]; b) polyhedral bodies called carboxysomes containing an enzyme needed for carbon fixation in autotrophic bacteria and similar enzymatic inclusions found in other bacteria [2,3,4]; c) storage granules that may or may not be surrounded by a membrane and which serve to store inorganic or organic compounds [5]; d) magnetosomes, membrane-bound iron-containing crystals present in some aquatic bacteria that respond to magnetic fields [6]; and e) amplified membranous structures that may or may not be continuous with the cell membrane, and which are visualized in photosynthetic and chemoautotrophic bacteria and are involved in processes of energy production [7,8].

Recent improvements in imaging techniques have allowed researchers to reassess bacterial architecture. Bacteria are no longer viewed as static and homogeneous cells, and the task of deciphering the structure, function and spatial organization of molecular machines inside the fluid architecture of bacterial cells has emerged as a new challenge [9,10]. Improvements in technical procedures and software have brought TEM studies closer to the native hydrated and three-dimensional (3D) state of biological specimens. The cryoimmobilization of specimens by rapid cooling followed by freeze substitution (FS) and TEM observation of sections of resin-embedded samples at room temperature, or freeze fracture (FF) and TEM observation of replica, or cryoelectron microscopy of vitreous sections (CEMOVIS) has avoided chemical fixation followed by room temperature dehydration and is far less likely to create artifacts [11]. Another step forward in the understanding of the 3D architecture and organization of bacterial cells and intracytoplasmic structures has been the study of 3D-reconstructions from tilt series. In electron tomography, a feature from a sample is imaged in the TEM at several angles by tilting the holder supporting the specimen-containing grid. In tomography, the sample can be an isolated specimen that does not need to be sectioned, or a structure inside a section. Specimen sectioning is frequently needed to analyze bacterial cytoplasmic structures, because the acquisition of high-quality TEM images is limited by sample thickness. Electron tomography can be performed using tilt series acquired at room temperature from sections obtained after cryoimmobilization by high-pressure freezing (HPF), FS, and resin embedding. The same 3D-reconstruction strategy can also be applied to tilt series acquired under cryoconditions (-170^°^C), on plunge-frozen samples or cryosections of high-pressure frozen samples. In recent years, many bacterial cells and known bacterial structures have been reanalyzed with improved TEM techniques, providing a new and more accurate view of the complex ultrastructure of prokaryotic cells [12,13,10].

The major technological advances in TEM have also opened up fresh opportunities for discovering new bacterial structures. New bacteria from unknown habitats constitute a particularly interesting subject of analysis in the quest for unusual features of prokaryotic cells [14,15]. Here we report the use of different TEM and cryo-TEM techniques for the structural examination of the new Antarctic bacterium *Pseudomonas* *deceptionensis* M1^T^ [16], revealing a cytoplasmic structure that, to our knowledge, has not been described before.

## Materials and Methods

### Cell growth

Studies were performed mainly with *Pseudomonas* *deceptionensis* M1^T^ (LMG 25555) isolated from marine sediment collected from Deception Island (Antarctica) and characterized by our group as a new species [16]. For most observations, using different techniques, samples of *P* *. deceptionensis* M1^T^ were grown for 12 days at 0^°^C on tryptone soy agar (TSA, Oxoid) according to the manufacturer’s specifications. Other experimental growing conditions were also tested, such as growing the strain on TSA for 6, 20 and 30 days at 0^°^C, or on TSA at 4^°^C, 15^°^C, 27^°^C at different times. Other growth media were also assayed such as BHI agar (Difco), Marine agar (Difco) or liquid minimal medium MM1 (g·l^-1^: glucose, 20; yeast extract, 0.1; NaNO_3_, 7; KH_2_PO_4_, 2; Na_2_HPO_4_·4H_2_O, 0.7; MgSO_4_·7H_2_O, 0.1; FeSO_4_ · 7 H_2_O, 0.018; trace elements, 1ml). *Pseudomonas psychrophila* DSM 17535^T^ and *Pseudomonas* *fragi* DSM 3456^T^ were grown on TSA (Oxoid) for 12 days at 0^°^C. *Pseudomonas fluorescens* ATCC 13430^T^ was grown on TSA (Oxoid) for 12 days at 10^°^C.

### High-Pressure Freezing (HPF)

HPF for samples to be freeze substituted was performed on colonies of *P* *. deceptionensis* grown on TSA plates which were transferred to 1.5 mm diameter and 200 µm depth planchettes without addition of cryoprotectants, immediately cryoimmobilized using a Leica EMPACT High-Pressure Freezer (Leica Microsystems, Vienna, Austria) and then stored in liquid nitrogen (LN_2_) until being freeze substituted [17].

HPF for samples to be cryosectioned was performed on colonies resuspended in 30% dextran (Fluka) in 0.01 M phosphate buffer saline (PBS). The suspensions were introduced into 350 µm inner diameter copper tubes and ultrarapid frozen using a Leica EMPACT High-Pressure Freezer (Leica Microsystems, Vienna, Austria). The copper tubes were stored in LN_2_ until further use for cryosectioning.

### Freeze Substitution (FS)

Planchettes containing the frozen samples were transferred, under LN_2_, to cryotubes containing the FS medium at -90^°^C in an EM AFS (Leica Microsystems, Vienna, Austria). For epoxy resin embedding, we used three different FS media with anhydrous acetone containing: 1. 1% osmium tetroxide (OsO4) (EMS, Hatfield, USA) and 0.25% glutaraldehyde (GA) (EMS, Hatfield, USA); 2. 1% OsO4, 0.1% uranyl acetate (UA) (EMS, Hatfield, USA) and 1% water [18]; and 3. 2% OsO4 and 0.1% GA. Samples were freeze substituted at -90^°^C for 48 h and warmed up to 4^°^C at a 5^°^C/h slope. Once the samples reached 4^°^C, they were kept at this point for 2 h and were transferred in darkness to room temperature for 2 h. The samples freeze substituted in 2% OsO4 and 0.1% GA were treated with an osmium-mediated tannic acid impregnation [19] (tannic acid, EMS, Hatfield, USA). All samples were infiltrated and embedded in Epon-812 resin (EMS, Hatfield, USA).

For acrylic resin embedding, samples were freeze substituted in 2% UA in acetone containing 1% water at -90^°^C for 1 hour, warmed up 20^°^C per hour until -50^°^C and kept at this temperature for 2 h. After several acetone rinses, samples were infiltrated with Lowicryl HM23 resin (EMS, Hatfield, USA) and polymerized under ultraviolet light for 48 h at -50^°^C followed by 48 h at 22^°^C [20].

### Room temperature sectioning

Sections of 60 nm in thickness were obtained using a UCT ultramicrotome (Leica Microsystems, Vienna, Austria), a 45^°^ diamond knife (Diatome, Biel, Switzerland) and a clearance angle of 6^°^. Epon sections were mounted on formvar coated 200 mesh copper grids and stained with aqueous or methanolic 2% UA and lead citrate. Lowicryl sections were mounted on formvar coated 200 mesh gold grids and stored until the immunolabeling process.

Sections of 250 nm in thickness were obtained in the same conditions as the ultrathin sections to perform electron tomographic studies. Sections were mounted on 200 mesh copper grids and stained with aqueous or methanolic 2% UA. Fiducial markers were then attached to both faces of the sections by successively floating them on drops of protein A coupled to 10 nm diameter colloidal gold particles (CMC, University of Utrecht, The Netherlands) 1:500 in 0.01 M PBS and distilled water.

### Tokuyasu technique for cryosectioning

*P* *. deceptionensis* M1^T^ colonies were collected with a loopful, resuspended in a chemical fixative containing 2.5% GA in 0.1 M PHEM pH 6.9 [0.06 M PIPES (Calbiochem), 0.025 M Hepes (Calbiochem), 0.01 M EGTA (Calbiochem), 0.002 M MgCl_2_ (Amresco)] and fixed for 2 h at room temperature. The suspension was centrifuged at 2000 rpm for 10 min and rinsed with 0.1 M PHEM. Pelleted cells were carefully resuspended in 12% gelatine (Merck) in 0.1 M PHEM, incubated for 5-10 min at 37^°^C, centrifuged to obtain a pellet and immediately placed on ice until the gelatine was hardened. Then, the pellet was cut into small pieces, which were infiltrated with 2.3 M sucrose (Fluka) in 0.1 M PHEM on a rotating wheel overnight at 4^°^C. Then, the small pieces were mounted onto sample pins and frozen in LN_2_. Pins with frozen samples were transferred to a pre-cooled (-100^°^C) EM FC6 cryoultramicrotome (Leica Microsystems, Vienna, Austria) covered with a homemade anticontamination glove box as explained below for vitreous sectioning.

Squared block faces with side measures ranging from 260 to 300 µm were trimmed using glass knives. Then, the temperature was lowered until -120^°^C and 60 nm cryosections were cut with a 35^°^ diamond knife (Diatome, Biel, Switzerland) with a clearance angle of 6^°^ and at cutting speeds between 0.3 and 1 mm/s. Ribbons were picked-up with a 1:1 mixture of 2% methyl cellulose (25 centipoises, Sigma-Aldrich) and 2.3 M sucrose and next, thawed and stored on formvar coated 200 mesh nickel grids (EMS, Hatfield, USA) at 4^°^C until the immunolabeling process.

### Vitreous cryosectioning

For vitreous cryosectioning, copper tubes coming from the HPF and stored in LN_2_ were transferred to a pre-cooled (-150^°^C) FC6 cryoultramicrotome (Leica Microsystems, Vienna, Austria) covered with a homemade anticontamination glove box [21]. An influx of dry nitrogen gas, dry sodium hydroxide pellets and dry silica gel balls was used to reduce the relative humidity within the chamber to values close to 0%. Squared block faces with side measures between 80 and 100 µm were trimmed from the copper tubes in black uniform areas from the sample, using a 45^°^ cryotrim diamond blade (Diatome, Biel, Switzerland). Then, 50 nm sections were cut with a 25^°^ diamond knife (Diatome, Biel, Switzerland) with a clearance angle of 6^°^ and at cutting speeds between 0.3 and 100 mm/s. Ribbons of vitreous sections were attached to Quantifoil^®^ carbon coated 200 mesh copper grids by electrostatic charging using the CRION (Leica Microsystems, Vienna, Austria). Once charged, the grids supporting the attached vitreous sections were transferred to grid boxes and stored in LN_2_ until being observed.

### DNA Immunolabeling

The grids containing Lowicryl HM23 ultrathin sections were rinsed in 0.01 M PBS and 0.05 M glycine (Amresco) in 0.01 M PBS. They were then incubated with blocking buffer by successively floating them on drops of 5% and 1% bovine serum albumin (BSA) in 0.01 M PBS solutions for 10 and 1 min, respectively. The grids were incubated 30 min at room temperature with monoclonal Mouse IgM Anti-ds-DNA antibody (Novus Biologicals, Littleton, USA, clone AC-30-10) diluted 1/10 in blocking buffer (1% BSA in 0.01 M PBS). After four washes on drops of 0.25% Tween 20 in 0.01 M PBS for 4 min and 1% BSA in 0.01 M PBS for 1 min, sections were incubated with IgM anti-mouse coupled to 12 nm diameter colloidal gold particles (Jackson, West Grove, USA) 1/30 diluted in blocking buffer for 15 min and rinsed with water. The grids were then rinsed in 0.01 M PBS, fixed in 1% GA in 0.01 M PBS and rinsed abundantly in distilled water. They were stained with 1% potassium permanganate and 1% UA in water for 15 min [22]. As a control for non-specific binding of the colloidal gold-conjugated antibody, the primary antibody was omitted*.*

Tokuyasu sections were incubated in 2% gelatine in 0.1 M PHEM for 30 min at 37^°^C and rinsed in 0.15 M glycine in 0.1 M PHEM. Then, the grids were incubated with blocking buffer by successively floating them on drops of 10% and 1% fetal bovine serum (FBS) in 0.1 M PHEM solutions for 10 and 2 min, respectively. They were incubated with monoclonal Mouse IgM Anti-DNA (Novus Biologicals, Littleton, USA, clone AC-30-10) 1/10 diluted in blocking buffer (1% FBS in 0.1 M PHEM) for 1 h. After eight washes with drops of 0.2% FBS in 0.1 M PHEM for 2 min and 1 with 1% FBS in 0.1 M PHEM for 2 min, sections were incubated with goat anti-mouse IgM coupled to 12 nm diameter colloidal gold particles (Jackson, West Grove, USA), 1/30 diluted in blocking buffer for 30 min. They were rinsed in 0.1 M PHEM, fixed with 1% GA in 0.1 M PHEM and abundantly rinsed with water. The grids were then stained with 2% uranyl oxalate [2% UA and 0.15 M oxalic acid (Fluka); pH 7] and 0.4% UA in 2% methylcellulose.

### Freeze Fracture (FF)

TSA-grown cultures of *P* *. deceptionensis* M1^T^ were sandwiched between two copper platelets using a 400 mesh gold grid as spacer and directly frozen by plunging in liquid propane at -189^°^C and fractured at -100^°^C and 10^-7^ mbar in a Bal-Tec BAF-060 freeze-etching system (Leica Microsystems, Vienna, Austria). The fracture was followed by freeze etching at -100^°^C for 2 min. The replicas were obtained by unidirectional shadowing of the exposed surface with 2 nm of Pt/C at 45^°^ and 20 nm of C at 90^°^ and were then floated on household bleach for 3 h, washed with distilled water and, finally, mounted on formvar coated 200 mesh copper grids.

### Imaging

Sections and replicas were observed in a Tecnai Spirit microscope (EM) (FEI, Eindhoven, The Netherlands) equipped with a LaB_6_ cathode. Images were acquired at 120 kV and room temperature with a 1376 x 1024 pixel CCD camera (FEI, Eindhoven, The Netherlands).

Electron tomography studies of semithin sections were performed at 120 kV, using the Tecnai Spirit EM described before or at 200 kV using a Tecnai F20 EM (FEI, Eindhoven, The Netherlands) equipped with a field emission gun and a 4096x4096 pixel CCD Eagle camera (FEI, Eindhoven, The Netherlands). Double axis tilt series were collected using the Xplore3D (FEI, Eindhoven, The Netherlands) acquisition program. The angular tilt range was typically set from -60^°^ to +60^°^ with a 1.5-2^°^ tilt increase.

Vitreous cryosections were transferred to Tecnai F20 using a cryoholder (Gatan, Warrendale, USA). Images were taken at 200 kV, at a temperature ranging from -175 to -170^°^C and using low-dose imaging conditions with the 4096x4096 pixel CCD Eagle camera. Electron diffraction was used to check whether water was vitreous or crystalline; crystalline sections were discarded.

### Statistical analysis

The one-factor ANOVA test was used to analyze measurements of stacks. Significance was set at P<0.05. The analyses have been carried out using Statgraphics software (version 5.1).

### Postprocessing of images and 3D Reconstructions

Micrographs from vitreous cryosections were denoised using the ImageJ 4.5.3 software by applying sequentially a 2.0-pixel-radius Median filter and a 2.0-sigma-radius Gaussian Blur filter.

The tilt series from Epon and Lowicryl sections were aligned using 10 nm gold fiducial markers on the two surfaces of the sections using the IMOD 4.5.3 software [23]. Then, the individual aligned series from dual axis tomography were separately reconstructed by the simultaneous iterative reconstruction technique (SIRT) using the Tomo3D package, version of April 2012 [24,25]. Tomograms were combined into dual axis tomograms using the IMOD package and denoised using the bilateral filtering (bbif) included in the Bsoft 1.8.2 software [26] or the Tomobflow software version of July 2011 [27]. Finally, dual axis tomograms were analyzed using IMOD (xyz slicer).

## Results

**Figure 1:**
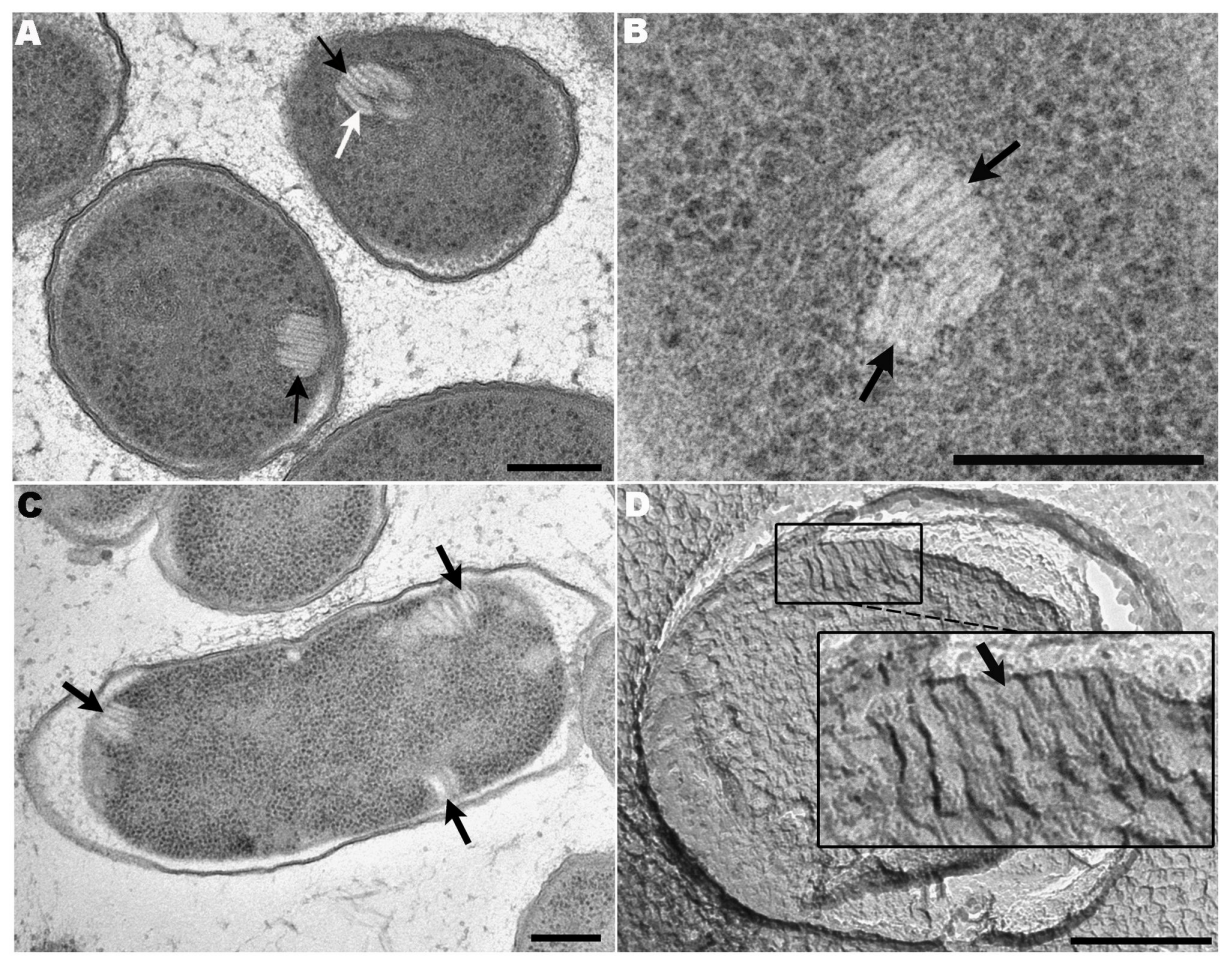
TEM micrographs of organized structures (stacks) located in the cytoplasm of TSA-grown cultures of *P* *. deceptionensis* M1^T^. (A–C) 60 nm Epon sections of samples processed by HPF-FS. (A) Elongated clustered parallel sticks located perpendicular to the PM (black arrows). Most sticks are visualized as straight units, but a slight curvature is observed in some of them (white arrow). (B) Two contiguous and differently oriented stacks in the bacterial cytoplasm (black arrows). (C) Three stacks perpendicular to the PM at different locations within the cytoplasm of a cell (black arrows). (D) A replica micrograph from a propane cryoimmobilized, FF and shadowed sample. A stack is observed perpendicular to the PM and comprised by about 8 sticks (see black arrow in the magnified area). Scale bars = 250 nm.

*Pseudomonas* *deceptionensis* M1^T^ is a new bacterium isolated from marine sediment collected on Deception Island, in the Antarctic area. The cells of this species have been described as rod-shaped, catalase- and oxidase-positive, and motile by means of a polar flagellum. Moreover, *P* *. deceptionensis* M1^T^ is a psychrotolerant strain able to grow at temperatures ranging from -4 to 34^°^C. A sample of *P* *. deceptionensis* M1^T^ grown on TSA plates at 0^°^C for 12 days was frozen by HPF without addition of cryoprotectants, and then freeze-substituted, Epon-embedded and sectioned for observation by TEM. The 60 nm sections revealed a highly organized structure located in the bacterial cytoplasm, which was unlike any cytoplasmic inclusion or structure reported to date. These structures, observed in two dimensions in the bacterial sections, comprised assemblies of a variable number of elongated parallel sticks, in most cases perpendicular to the plasma membrane (PM) (Figure 1A, black arrows). Most of the clustered sticks were straight, but some were slightly curved (Figure 1A, white arrow). Henceforth, we have used the term “stack” to refer to each complete assembly of stick-like shapes.

To study the stacks in greater detail, we first counted the sticks in each one. This was done with 170 stacks observed in micrographs of Epon sections of *P* *. deceptionensis* M1^T^. A total of 854 sticks were counted, with a mean value of 5.02 per stack. However, a significant dispersion was observed, with sticks ranging from one to 14 per stack (Table 1). From the same micrographs we measured the length and width of 59 sticks (Table 2, first row). The mean length was 93.71±26.22 nm, but the values ranged widely from 39.65 to 160.59 nm. In contrast, stick width was much more homogeneous, with a mean value of 15.88±2.25 nm.

**Table 1:** Number of sticks per stack (N) observed in 170 stacks from Epon sections of *P* *. deceptionensis* M1^**T**^ cells processed by HPF-FS.

N	Frequency	%
1	4	2.35
2	20	11.76
3	27	15.88
4	28	16.47
5	26	15.29
6	24	14.12
7	15	8.82
8	14	8.24
9	3	1.76
10	5	2.94
11	1	0.59
12	2	1.18
14	1	0.59

**Table 2:** Mean values of length and width of the sticks according to different TEM techniques.

	Mean Length	SD	Mean Width	SD
HPF + FS + Epon	93.71	26.22	15.88	2.25
FF	75.03	11.54	13.59	2.14
HPF + FS + HM23	85.85	22.46	12.81	1.69
Tokuyasu	85.49	19.04	14.13	1.73
CEMOVIS	76.35	23.68	12.09	3.17

(SD) Standard deviation; (HPF + FS + Epon) High-pressure freezing, freeze substitution and Epon; (FF) Freeze-fracturing; (HPF + FS + HM23) High-pressure freezing, freeze-substitution and HM23 Lowicryl; (CEMOVIS) Cryoelectron microscopy of vitreous sections.

Interestingly, stacks were only frequently observed in *P* *. deceptionensis* M1^T^ cells grown under specific growth conditions (on TSA plates for 12 days at 0^°^C): 23.23% of 452 *P* *. deceptionensis* M1^T^ cells counted from Epon ultrathin sections showed stacks in their cytoplasm. In contrast, when using shorter or longer incubation times or higher incubation temperatures, stacks were found only sporadically. Furthermore, we rarely observed stacks in cells grown in liquid media and, therefore, we were unable to determine whether the presence of stacks was related to a certain stage of the growth curve.

Micrographs of the strain also revealed that *P* *. deceptionensis* M1^T^ cells seemed to contain one or more stacks at the same time. Most *P* *. deceptionensis* M1^T^ cells observed in Epon ultrathin sections showed one or two stacks (Table 3). The quantifications suggested that each cell may contain at least between one and four stacks, although obviously this is not a definitive measurement of the total number since only 60 nm cell sections were visualized.

**Table 3:** Number of stacks per 60 nm cell section (N) observed in 452 cells from Epon ultrathin sections of *P* *. deceptionensis* M1^**T**^ processed by HPF-FS.

N	Frequency	%
0	347	76.77
1	72	15.93
2	27	5.97
3	4	0.89
4	2	0.44

When more than one stack appeared simultaneously in the cytoplasm, TEM observation of Epon sections showed that the stacks were variable positioned: either close together (Figure 1B, black arrows) or scattered in different locations (Figure 1C, black arrows), the former being observed twice as frequently.

Stacks were also observed after FF, which provided a replica of the rough fractured surfaces after ultra rapid freezing, in this case by plunge freezing in propane without any addition of chemical fixatives or cryoprotectants. The fracture of the frozen sample at -100^°^C was performed inside a high vacuum to avoid water condensation and keep all the cellular components in place. This technique was used to confirm that the stacks were not artifacts produced by the FS process, fixatives, or changes in temperature. Figure 1D depicts a clearly visible stack after FF, containing several parallel sticks perpendicular to the PM with a mean length of 75.03±11.53 nm and a mean width of 13.59±2.13 nm (Table 2, second row), which were similar to the values in samples obtained after HPF-FS and Epon-embedding.

**Figure 2:**
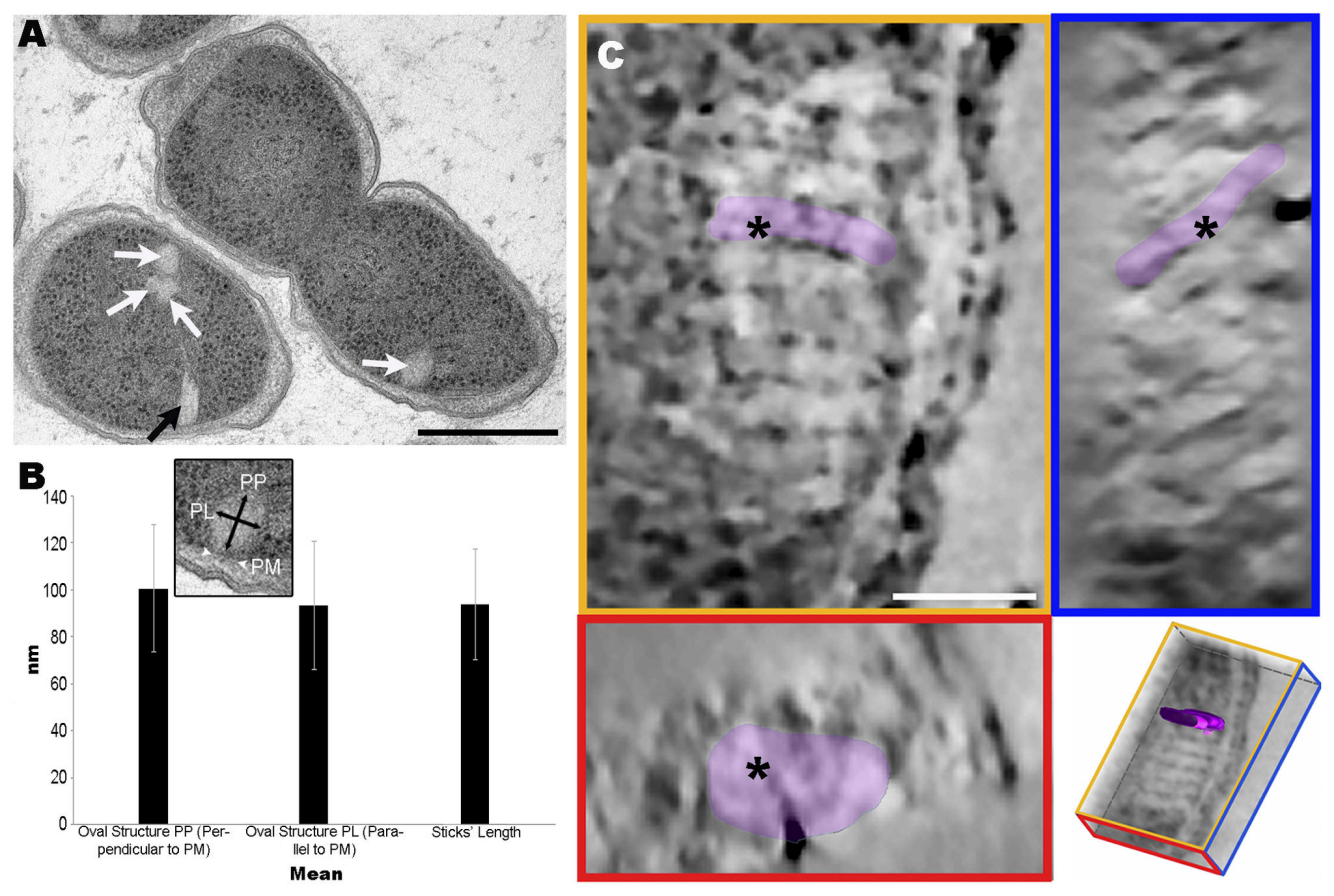
TEM analysis of oval structures observed in *P* *. deceptionensis* M1^T^ TSA-grown cultures processed by HPF-FS. (A) A 60 nm Epon section showing a stack (black arrow) and oval structures (white arrows). Scale bar = 250 nm. (B) Graph comparing the oval structure measurements (PP, PL) and the length of the sticks. The error bars correspond to standard deviation values (SD) (PP). Perpendicular; (PL) Parallel; (PM) Plasma membrane. (C) 4 nm tomogram slices from the XYZ views of a bbif-filtered tomogram reconstructed from 250 nm Epon sections. The asterisks correspond to the same point through the different views XYZ. The XY view (top-left image) reveals a stack perpendicular to the PM, in which one clustered unit has been colored. The YZ view (top-right image) shows the same stack in which sticks are distributed obliquely within the section. The dyed unit corresponds to the one colored before. The XZ view (bottom-left image) shows the same dyed stick observed as an oval structure. The bottom right picture corresponds to a scheme of the view’s distribution in the tomogram where the highlighted unit has been segmented. Scale bar = 50 nm.

Some *P* *. deceptionensis* M1^T^ cells grown for 12 days at 0^°^C and processed by HPF-FS and Epon embedding also presented oval structures (Figure 2A, white arrows), which were observed at similar cytoplasmic locations and frequencies as the stacks, and also with a perpendicular orientation to the PM. We took two measurements of each of these oval structures: one perpendicular and another parallel to the PM (Figure 2B). A total of 155 oval structures were measured, revealing a mean perpendicular length of 100.57±27.01 nm and a mean parallel length of 93.26±27.21 nm, values similar to those of the sticks (Figure 2B). Given the similarities in location, frequency and size between the oval structures and sticks, we wondered whether they could correspond to different 2D views of the same structure, that is, whether the stick shapes observed in the stacks may represent transversal sections of flattened oval structures.

Semithin sections of *P* *. deceptionensis* M1^T^ cells grown on TSA at 0^°^C for 12 days and processed by HPF-FS and Epon embedding were further explored by 3D electron tomography. Dual-axis tilt series from 250 nm Epon sections were acquired in the TEM at 120 kV, each tilt series being reconstructed using the Tomo3D software. Tomograms corresponding to each series were combined with the IMOD software and the final dual-axis tomogram was obtained (Movie S1). Figure 2C shows the XY, ZY and XZ tomogram slices from a point within the dual-axis tomogram observed in Movie S1 (the point is marked by an asterisk through the different views). The XY view (top left image) shows a stack composed of 10 elongated parallel sticks with a perpendicular orientation to the PM. The YZ view (top right image) shows an oblique orientation of the same flat structures within the semithin section. Finally, the XZ view (bottom left image) reveals oval structures like those observed in the ultrathin sections. This result confirms that the stick and oval shapes were in fact different 2D projections of the same structure, and in 3D the stacks consisted of groups of parallel oval discs. Henceforth, the units composing the stacks will be referred to interchangeably as “discs” or “sticks”.

**Figure 3:**
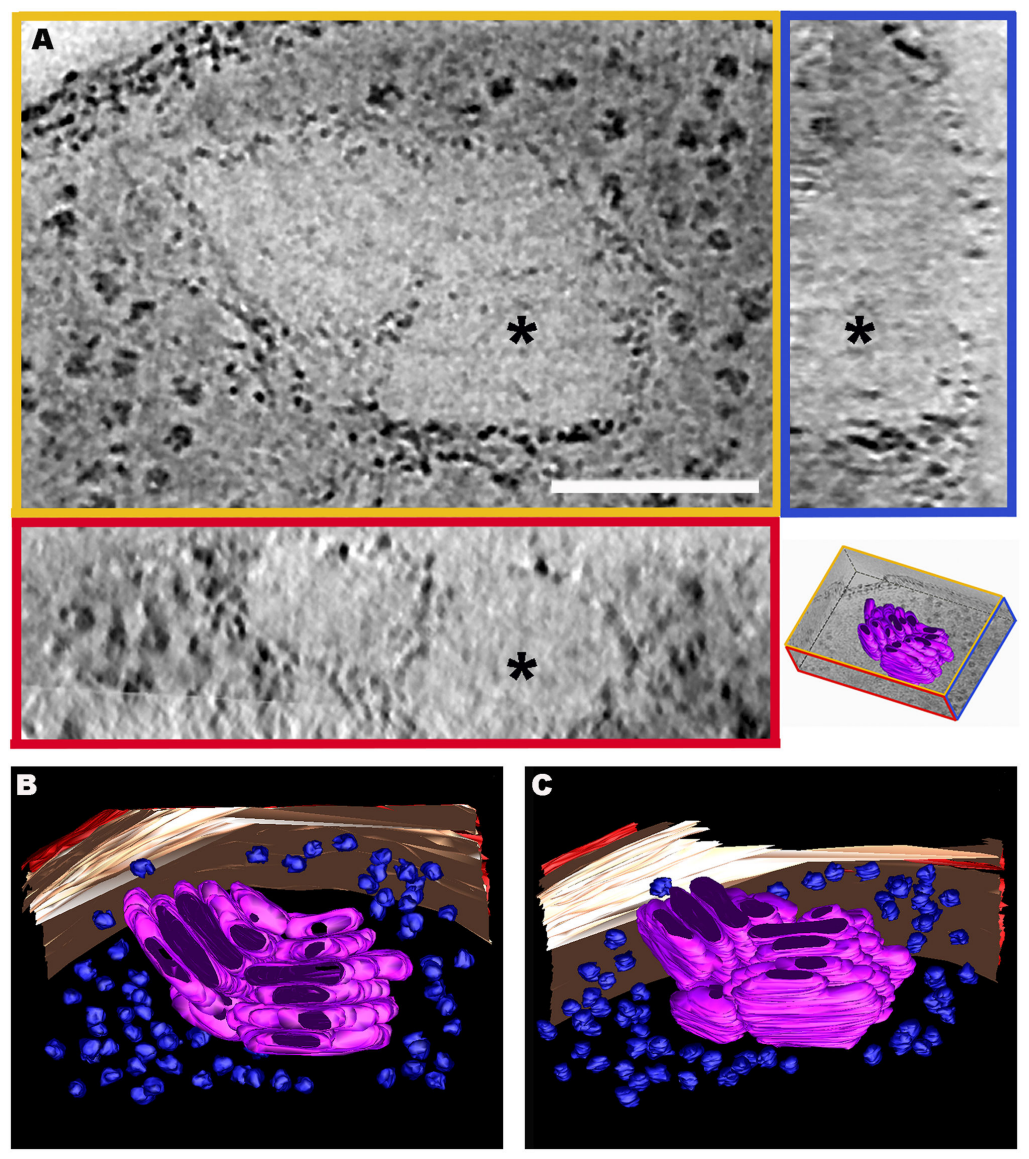
3D visualization of stacks observed in *P* *. deceptionensis* M1^T^ cells after HPF-FS. (A) 2 nm tomogram slices from the XYZ views of a bbif-filtered tomogram reconstructed from a 250 nm Epon section. The asterisks correspond to the same point through the different views. Scale bar = 100 nm. The XY view (top-left image) shows a peripheral portion of the cytoplasm of a cell where two contiguous stacks can be visualized. The YZ view (top right image) shows the clustered sticks from the stack on the right in the XY view. The XZ view (bottom left image) reveals two stacked units as oval structures. The bottom right picture corresponds to a scheme of the view’s distribution in the tomogram. (B–C) Two different views from the segmentation of the tomogram observed in (A), which reveal the 3D structure of the stacks within the tomogram as groups of oval discs. In red, the outer membrane; in cream-color, the PM; in blue, the ribosomes; and in pink, the discs.

The reconstruction observed in Movie S2 was performed in the same conditions as in Movie S1, but using tilt series captured at 200 kV. Figure 3 shows the XY, ZY and XZ tomogram slices from the same point (the point is marked by an asterisk through the different views) of the double-axis tomogram presented in Movie S2 (A), and two views of its segmentation (B and C). The XY view in Figure 3A (top left image) clearly shows two contiguous stacks at an angle of 130^°^ to each other. Both are located near the boundaries of the cell PM and the one on the left is perpendicular to the PM. In the YZ view, the stack shown appears as a pile of flat structures oriented perpendicularly to the surface (top right image). The XZ view (bottom left image) shows two oval structures corresponding to the frontal views of two flat discs. The tomogram segmentation from the dual-axis tomogram observed in Movie S3 and Figure 3B and 3C confirm the presence of two contiguous stacks, one on the right and one on the left, formed by parallel oval discs. In the segmentation process, the outer membrane is colored red, the PM cream, ribosomes blue, and the oval discs of the stacks are pink.

**Figure 4:**
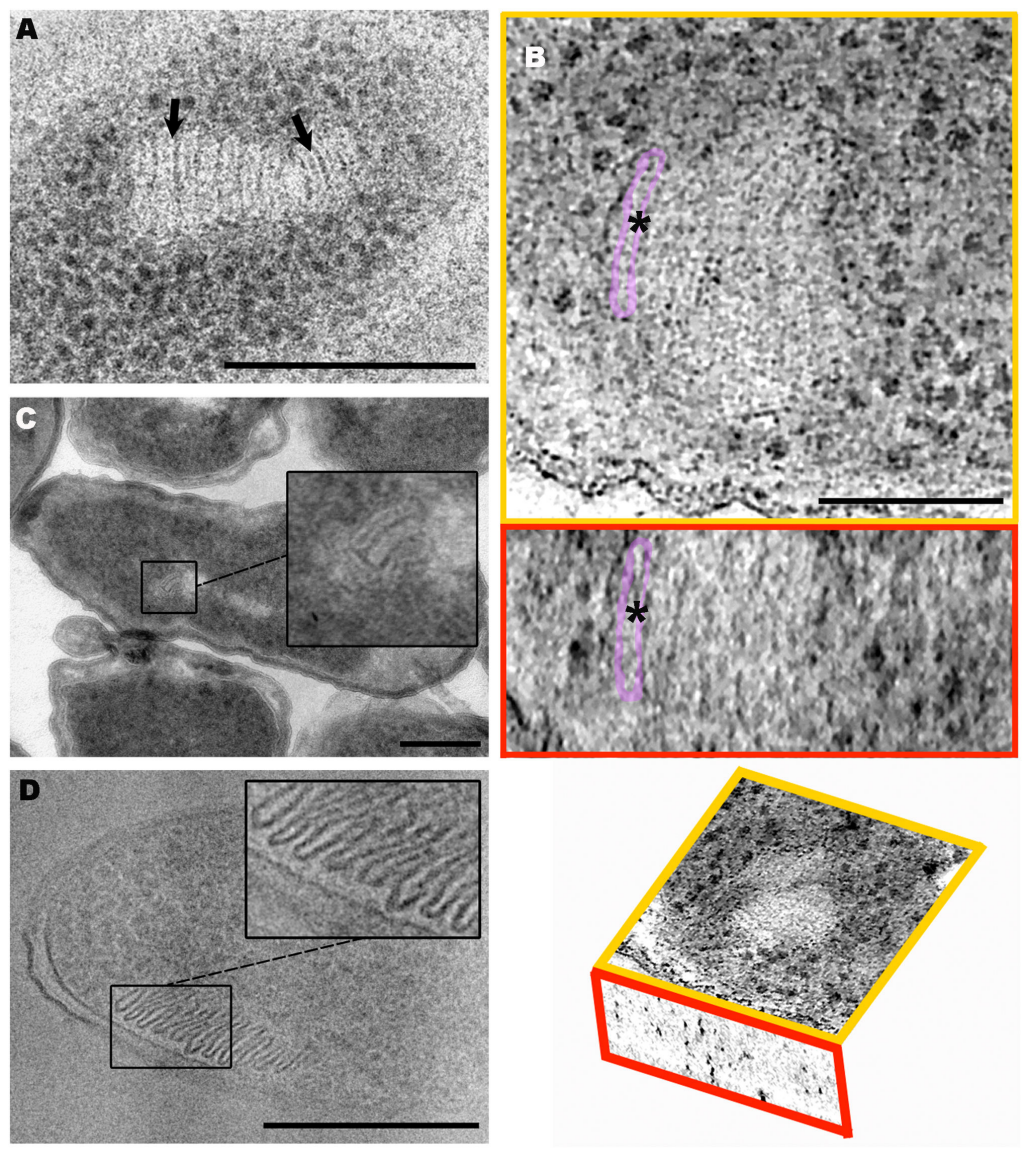
TEM and Cryo-TEM analysis of stacks of *P* *. deceptionensis* M1^T^. (A) A 60 nm Epon section from a sample processed by HPF-FS. A stack is visualized composed by clustered discs transversally cut, each of which is delimited by a membrane-like structure (black arrows). Scale bar = 250 nm. (B) 1.5 nm tomogram slices from the XYZ views of a bflow-filtered tomogram reconstructed from 250 nm Lowicryl HM23 sections. The asterisks correspond to the same point through the different views. The XY (top image) and the XZ (middle image) views show flat clustered discs transversally cut and perpendicularly distributed to the PM in a fragment of a cell. A membrane-like structure is observed surrounding each flat disc, one of which has been colored in both views. The bottom picture is a scheme of the view’s distribution in the tomogram. Scale bar = 100 nm. (C) A 60 nm Tokuyasu section. Stacked discs are also observed well delimited by a membrane-like structure (see magnified squared area). Scale bar = 250 nm. (D) A 50 nm vitreous cryosection micrograph (CEMOVIS). Clustered discs are observed clearly delimited by a membrane (see squared area). Scale bar = 250 nm.

In the TEM observations of *P* *. deceptionensis* M1^T^ Epon sections, discs were frequently surrounded by a membrane-like structure (Figure 4A, black arrows). To shed more light on the composition of this membrane, new samples were processed by HPF-FS and Lowicryl HM23 embedding or by the Tokuyasu method. Both techniques have been reported to improve membrane visualization, the latter clearly distinguishing lipid membranes.

Lowicryl HM23 sections allowed the visualization of stacked structures with similar dimensions to those previously described using other techniques. We measured 90 sticks, obtaining a mean length of 85.85±22.46 nm and a mean width of 12.81±1.69 nm (Table 2, third row). To study the membrane-like structures surrounding the discs, we analyzed tomograms from 250 nm Lowicryl HM23 sections reconstructed from tilt series acquired at 200 kV (Movie S4). Figure 4B shows the XY and XZ tomogram slices from the same point within the tomogram presented in Movie S4 (the point is marked by an asterisk through the different views). The XY view shows a stack perpendicular to the PM, with discs surrounded by membrane-like structures (highlighted in top image of Figure 4B), which also appear in the XZ view (middle image of Figure 4B showing the same highlighted structure as in the XY view).

The Tokuyasu technique also revealed the presence of stacks in the cytoplasm of *P* *. deceptionensis* M1^T^ cells, which appeared as elongated sticks (Figure 4C, squared area). After measuring 28 sticks, a mean length of 85.49±19.04 nm and a mean width of 14.13±1.73 nm were obtained (Table 2, fourth row). The micrographs revealed that the sticks were delimited by a white membrane-like structure with a similar electron density to the PM.

Cryoelectron microscopy of vitreous sections (CEMOVIS) was also performed in *P* *. deceptionensis* M1^T^ cultures grown on TSA at 0^°^C for 12 days to study the strain ultrastructure in a close-to-native state. The images of 50 nm vitreous cryosections showed stacks distributed perpendicularly to the PM, coinciding with the previous descriptions using other techniques. We measured 95 sticks, obtaining a mean length of 76.35±23.68 nm and a mean width of 12.09±3.17 nm, similar to the values obtained with other methods (Table 2, fifth row). Vitreous cryosections also revealed that the discs were delimited by a membrane-like structure that seemed to have a similar electron density to the PM of the cell (Figure 4D).

The perpendicular orientation of the stacks in close proximity to the PM, together with the similarities in the membranous structures identified by HPF-FS and Lowicryl HM23 embedding, the Tokuyasu method, and CEMOVIS, suggested that the stacks might be invaginations from the PM. Nevertheless, none of the techniques showed any continuity between disc membranes and the PM. Furthermore, when both membranous structures were clearly visualized, the PM appeared straight and uninterrupted, without any visible connections with the membrane surrounding the stacks (Figure 4D and Figure S1). In addition, we took 170 measurements of PM and disc membrane thickness from 34 bacteria. All the measurements were done on micrographs of vitreous cryosections, since this technique creates the fewest distortions at a molecular level. Mean values were 4.45±1.25 nm for the PM of the cell and 3.38±0.98 nm for the membrane surrounding the discs. After applying a one-factor ANOVA test between the two series of data, a p-value of 0.0000 was obtained, indicating significant differences between them. This result supported the idea that stacks were independent structures rather than invaginations of the PM.

**Figure 5:**
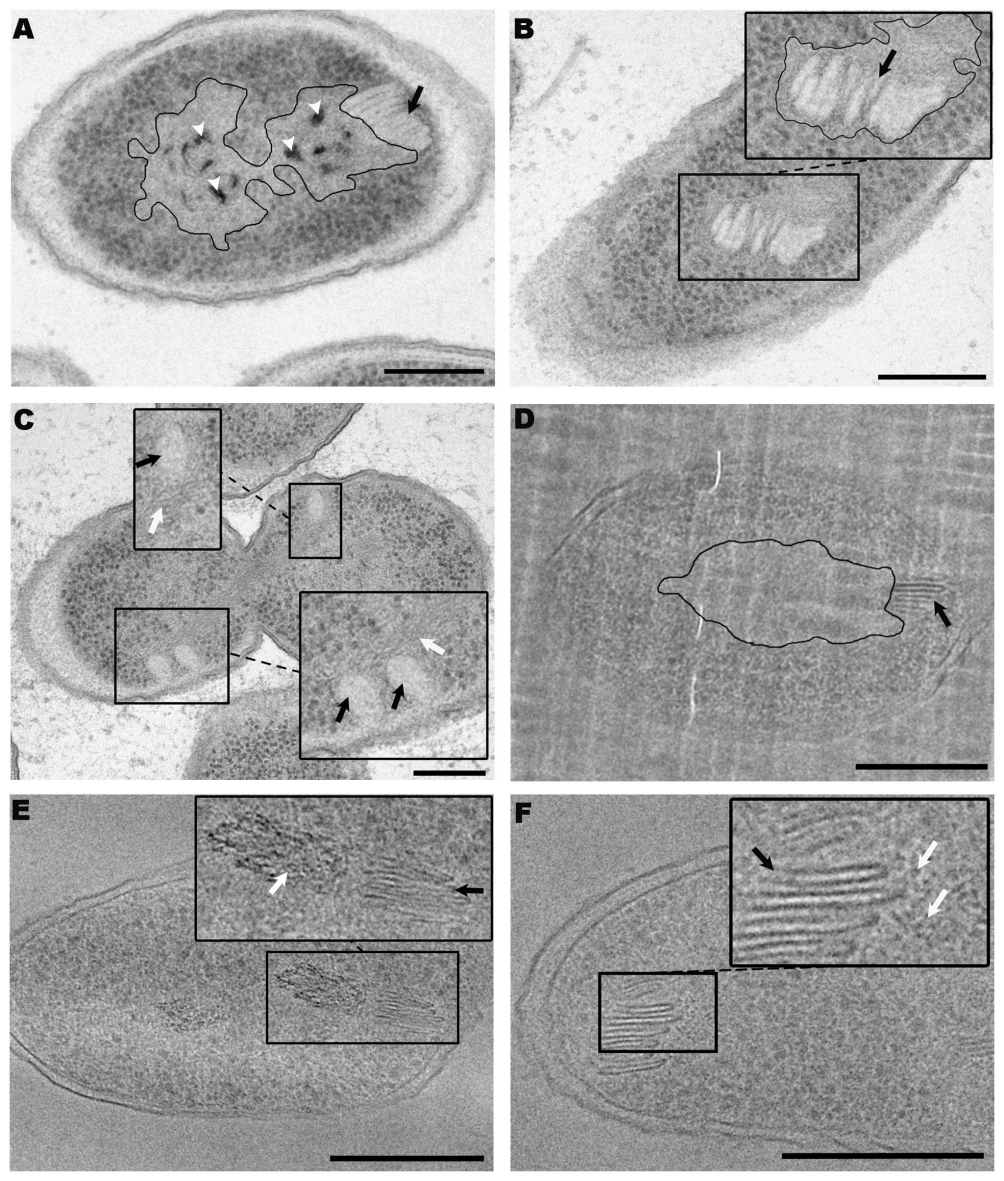
Study of the proximity between stacks and DNA observed in TSA-grown cultures of *P* *. deceptionensis* M1^T^ cells. (A–C) TEM micrographs of 60 nm Epon sections of samples processed by HPF-FS. (A) A stack (black arrow) perpendicular to the PM and very close to the bacterial nucleoid (outlined area). The bacterial nucleoid shows dark spots, corresponding to poly P granules (white arrow heads). (B) A stack (black arrow) embedded in the nucleoid area (outlined area) is shown. (C) A *P* *. deceptionensis* M1^T^ dividing cell distributing its DNA between its daughter cells is observed, in which stacks (black arrows) are visualized very close to the DNA (white arrows). (D–F) Cryo-TEM micrographs of 50 nm vitreous cryosections (CEMOVIS). (D) A stack (black arrow) is observed very close to a ribosome free area (RFA), corresponding to the nucleoid area (outlined area). (E) A stack (black arrow) is placed in the vicinities of a locally ordered arrangement of DNA microfibers (white arrow). (F) A stack (black arrow) is visualized very close to DNA microfibers (white arrows). Scale bars = 250 nm.

Interestingly, stacks observed near the periphery of HPF-FS Epon-embedded cells grown at 0^°^C for 12 days frequently appeared very close to DNA microfibers from the nucleoid (Figure 5A, nucleoid outlined and stack marked by black arrow), in some cases being completely embedded (Figure 5B, nucleoid outlined and stack marked by black arrow). In cells observed dividing and distributing their DNA among daughter cells, stacks were also visualized very close to the DNA fibers (Figure 5C, DNA microfibers marked by white arrows and stacks by black arrows). Inorganic polyphosphate (Poly P) granules were commonly observed in nucleoid areas located close to a stack (Figure 5A, white arrow heads).

The same proximity between DNA and stacks described in Epon sections was observed in vitreous cryosections. Stacks were localized next to ribosome-free areas (RFA), corresponding to the bacterial nucleoid (Figure 5D, nucleoid outlined and stack marked by black arrow). Furthermore, locally ordered arrangements of DNA and DNA microfibers were observed very close to the stacks (Figure 5E and Figure 5F, DNA marked by white arrows and stacks by black arrows).

**Figure 6:**
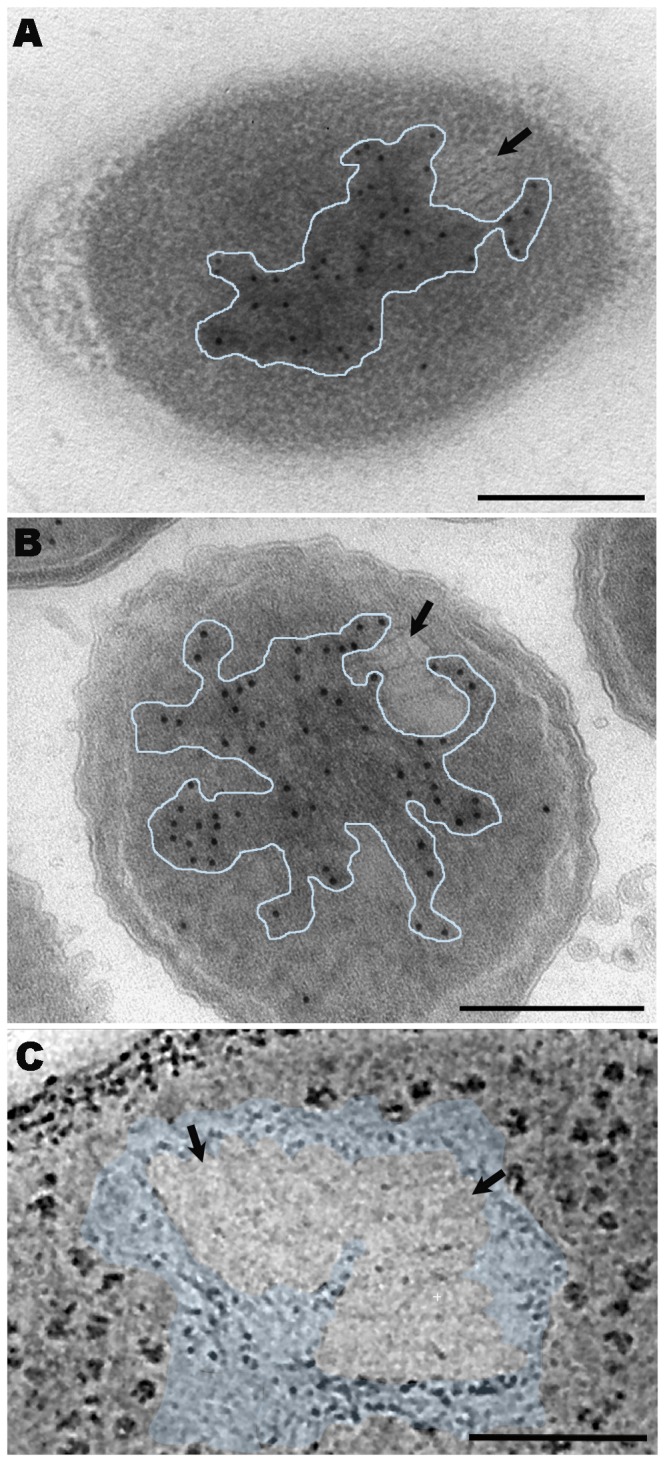
TEM immunolabeling and tomographic studies analyzing the proximity between stacks and DNA in TSA-grown cultures of *P* *. deceptionensis* M1^T^. (A) DNA immunolabeling on 60 nm HM23 Lowicryl sections from samples processed by HPF-FS. (B) DNA immunolabeling on a 60 nm Tokuyasu sections. (A–B) Stacks were observed very close to and partially embedded in to the gold-labeled nucleoid area (see black arrows pointing stacks and outlined areas corresponding to the nucleoids). (C) A 2 nm tomogram slice of a bbif-filtered tomogram reconstructed from a 250 nm Epon section of a sample processed by HPF-FS. Two contiguous stacks are observed (black arrows) embedded in the nucleoid area (see colored area). Scale bars = 250 nm.

To study the hypothetical relation between DNA microfibers and stacks in more detail, DNA immunolabeling and tomographic studies were performed on samples of *P* *. deceptionensis* M1^T^ TSA-grown at 0^°^C for 12 days. Immunolabeling experiments were performed in HM23 and Tokuyasu sections using a specific antibody for labeling double stranded DNA. In the HM23 sections, we also amplified the signal obtained with a DNA staining method using potassium permanganate, which allows the chromatin distribution within the bacterial cytoplasm to be visualized. The micrographs again revealed stacks at the periphery of the cytoplasm, contiguous to or partially embedded in DNA microfibers (Figure 6A and Figure 6B, black arrows mark the stacks and the nucleoid is outlined in blue). Electron tomographic studies of Epon sections of *P* *. deceptionensis* M1^T^ cells also showed stacks completely embedded in DNA microfibers along the Z-axis (see Movie S2 and Figure 6C, black arrows mark the stacks and the nucleoid is colored in blue).

**Figure 7:**
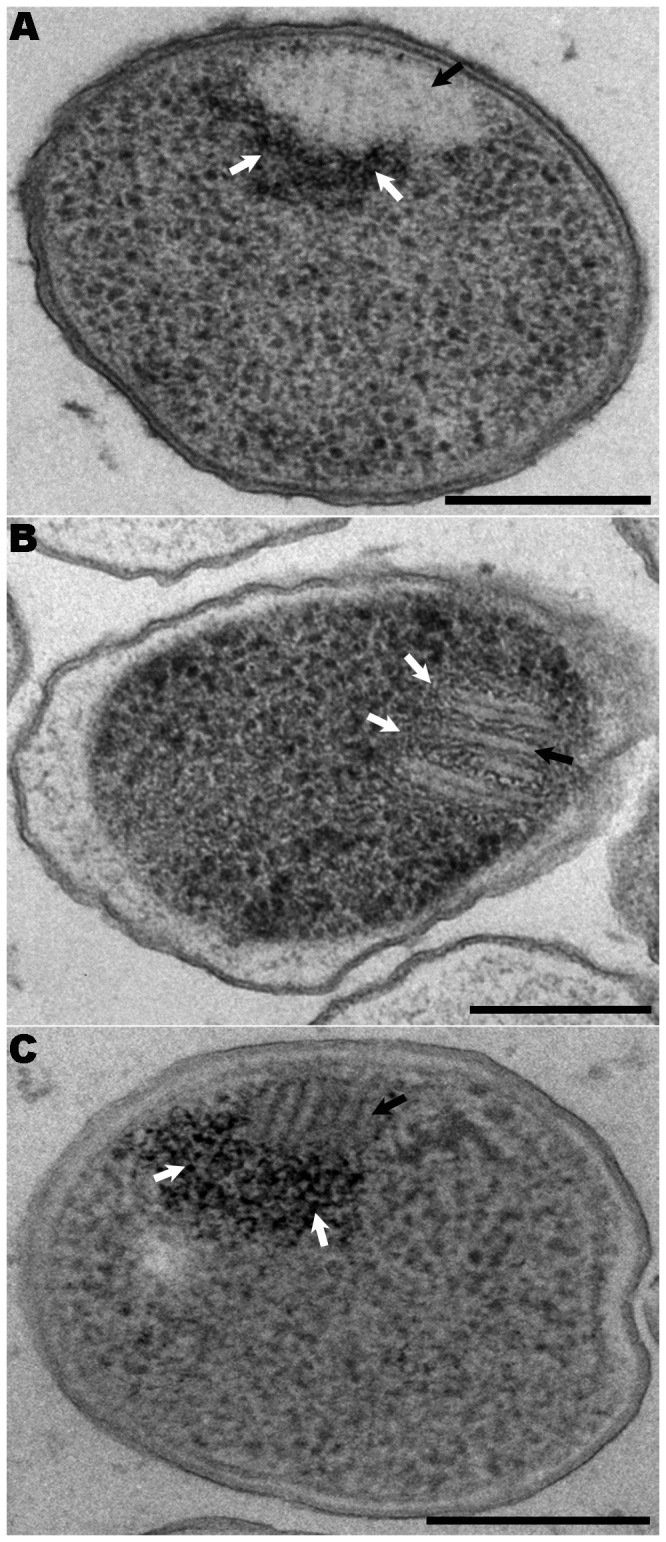
Stacks visualized in different *Pseudomonas* species from samples processed by HPF-FS. (A–C) 60 nm Epon sections. (A) *P. psychrophila* DSM 17535^T^ TSA-grown culture. (B) *P* *. fragi* DSM 3456^T^ TSA-grown culture. (C) *P. fluorescens* ATCC 13430^T^ TSA-grown culture. (A–C) The three strains show stacks perpendicularly distributed to the PM (see black arrows) very close to DNA microfibers (see white arrows). Scale bars = 250 nm.

As the stacks appeared to be novel structures, we wanted to study whether their presence was exclusive to the new Antarctic bacterium *P* *. deceptionensis* M1^T^ or whether they were general bacterial structures. We chose three bacterial species within the *Pseudomonas* genus, two of which, *P. psychrophila* DSM 17535^T^ and *P* *. fragi* DSM 3456^T^, are closely related to *P* *. deceptionensis* M1^T^, and another one, *P. fluorescens* ATCC 13430^T^, is phylogenetically more distant. The range of growth temperatures was determined for each species in order to reproduce slow-growing conditions. The samples were then processed by HPF-FS and Epon-embedding and imaged with TEM. Micrographs revealed that *P. psychrophila* DSM 17535^T^ and *P* *. fragi* DSM 3456^T^ incubated at 0^°^C and *P. fluorescens* ATCC 13430^T^ incubated at 4^°^C for 12 days all had stacks in their cytoplasm. In these three bacteria, the stacks were also found perpendicular to the PM, and close to DNA microfibers presenting inorganic polyphosphate, as previously described for *P* *. deceptionensis* M1^T^ (Figure 7A, 7B and 7C, respectively; stacks marked by black arrows and DNA microfibers by white arrows).

## Discussion

Thanks to the great advances in TEM and Cryo-TEM methodologies [28,29,30], we were able to visualize and analyze an unusual intracytoplasmic structure in the new Antarctic bacterium *Pseudomonas* *deceptionensis* M1^T^. To our knowledge, this structure has not been described before and does not correspond to any of the inclusions, membrane formations or other structures previously identified as components of bacterial cytoplasm. Depending on the technique used and the plane of the sample section, the new structure was visualized as a set of stacked parallel sticks of variable length, or as oval structures normally perpendicular to the cytoplasmic membrane. Tomograms then showed that these new structures are composed of a variable number of discs of various sizes, each one surrounded by a membrane-like envelope. We have used the term “stacks” to describe these structures.

When analyzing bacterial structures by TEM, a risk to be born in mind is the possible generation of artifacts that may be confused with structural elements, or lead to a misinterpretation of the structure. This has happened in the past when using conventional TEM techniques such as chemical fixation and room temperature dehydration. Two of the most common artifacts reported have been mesosomes, consisting of foldings of the PM in contact with the nucleoid, and rope-like DNA fibrils, which accumulate in the cytoplasm [31,32,33]. We initially observed stacks in *P* *. deceptionensis* M1^T^ cells processed by HPF-FS and Epon embedding, an approach known to allow good preservation of all components of bacterial cells, although it cannot unambiguously establish whether the ultrastructure is completely preserved at the molecular level. To confirm the existence of these new structures we used the main techniques currently available for thin-section analysis. We were also able to visualize stacked sticks or discs in samples processed by HPF-FS followed by Lowicryl HM23 resin embedding, and in tomography of plastic sections, Tokuyasu cryosections, and vitreous cryosections (CEMOVIS) [34,35,36,37,30]. The new structures were also observed with the FF technique, which offers a complementary perspective to thin-section analysis [38].

Although CEMOVIS is now accepted as the best method for preserving and analyzing bacterial structures, other techniques can provide complementary information [39]. Notably, there was little difference in the visualization of stacks among techniques, which gave very similar mean values of stick length and width (though the length showed greater variability). In addition, the tomograms demonstrated that the stacked structures were in fact not composed of stick-shaped forms, as initially observed, but flat discs.

The HM23, Tokuyasu and vitreous sections confirmed that the discs comprising each stack are surrounded by a membrane-like structure, although its composition could not be unequivocally assessed with these techniques. The membrane observed with CEMOVIS presented a similar profile to the cytoplasmic membrane, but significant differences in the mean width indicated variations in composition or structure. In Tokuyasu cryosectioning, which outperforms other techniques with regard to membrane visibility, the white staining of the discs membrane resembled that of cell lipid envelopes, which may indicate that this membrane is lipid in nature. The presence of a membrane surrounding the discs was also suggested by the FF-TEM analysis, although its appearance did not correspond to the cell bilayer membrane structure, since no characteristic globular intramembranous proteinaceous particles were seen attached to the fractured stick surfaces. Thus, our data suggest that the stacks are surrounded by a membrane that is not continuous with the PM, but we cannot yet confirm its composition or nature.

These observations ruled out the possibility that the newly described structures are artifacts, such as the case of mesosomes. The latter appear when bacteria are chemically fixed before any other process, causing certain membrane lipids to diffuse and assemble in the mesosome structure [40], a controversy eventually resolved by CEMOVIS and FS studies [41]. In our study, even the Tokuyasu technique (which also starts with a chemical fixation) preserved the stacks, since the state created by the chemical fixation was maintained throughout the subsequent low temperature procedures.

Two other significant features of the stacks are their variability and the fact that they were only visualized with frequency when the strain was grown at temperatures near the minimum required for growth (-4^°^C in the case of *P* *. deceptionensis* M1^T^). These structures varied in the number of stacks per cell, the number of discs in each stack, the size of the discs and the location of the stacks within the cell. Such variability suggests that they may be dynamic structures that are required to localize certain molecules in a particular place to perform a particular cellular function.

The fact that the stacks were mainly observed at 0^°^C may be associated with the slow growth of *P* *. deceptionensis* M1^T^ at this temperature, which may prolong dynamic processes, making it easier to capture the temporary structures involved. In addition, TEM and Cryo-TEM based on cryoimmobilization by HPF allowed us to fix cells in terms of milliseconds and thus reveal specific cellular moments when structures are quickly assembled in the place where their function is required, to be then dismantled once their function is fulfilled. Since the stacks were not visualized in samples processed by conventional preparation for TEM or in rapid-growing cells, we hypothesize that they may constitute a kind of dynamic structure that would have been untraceable before the use of slowed-down growth cultures and cryopreparation techniques.

Despite their variability, an interesting finding is that the stacks observed in most of the preparations were embedded in or very close to DNA fibers. This was clearly visualized in Epon sections from samples processed by HPF-FS and in vitreous cryosections (CEMOVIS), the latter technique providing the best observations of the bacterial DNA structure without aggregation artifacts [42]. This suggests a possible relationship between these new structures and certain processes involved in the bacterial chromosome dynamics. However, we were unable to analyze this correlation, for two main reasons. Firstly, we could not visualize stacks in sections from cells grown on liquid media, which would have allowed us to use the cell synchronization methods required for the study of chromosome dynamics and cell cycles in bacteria [43,44,45,46]. Secondly, we have not yet found a way of displaying these structures frequently enough in model bacteria, such as *E. coli* or *B. subtilis*, to perform studies of this kind (data not shown).

It should be emphasized that stacks are not exclusive to the new Antarctic bacterium *P* *. deceptionensis* M1^T^. We have visualized them clearly in other species of the *Pseudomonas* genus, where they were structurally very similar, seen only in slow-growing cells, and also observed close to DNA fibers.

To sum up, applying TEM cryotechniques to study the new Antarctic bacterium *Pseudomonas* *deceptionensis* M1^T^ and other bacteria in slow-growing conditions, we have demonstrated the existence of a new cytoplasmic structure, which for the moment can be described as a set of stacked membranous discs arranged perpendicularly to the PM but not continuous with it. This interesting new structure merits further cryoelectron tomography studies to define its configuration more precisely. Furthermore, once the conditions for its visualization in model bacteria are identified, it will be possible to explore if it is associated with chromosome dynamics [47].

## Supporting Information

Figure S1 **TEM visualization of stacks from *P* *. deceptionensis* M1^**T**^ cells processed by HPF-FS.** (A–D) 60 nm Epon sections. The PM is observed straight and uninterrupted and no continuity with stacks is observed in any case. Scale bars = 250 nm.
(TIF)

Movie S1 **A dual-axis tomogram from a 250 nm Epon section.** The tilt series were acquired at 120 kV and the tomogram was filtered with bbif (Bsoft). A stack composed by 10 discs is observed perpendicular to the PM.
(ZIP)

Movie S2 **A dual-axis tomogram from a 250 nm Epon section.** The tilt series were acquired at 200 kV and the tomogram was filtered with bbif (Bsoft). Two contiguous stacks displaced at an angle of 130^°^ from each other are visible. Both are located close to the PM, and one of them is oriented perpendicular to the PM. Each stack is observed as a cluster of oval discs.
(ZIP)

Movie S3 **Segmentation of the tomogram presented in Movie S2.** The 3D structure of the stack is revealed within the tomogram as a group of oval flat discs. In red, the outer membrane; in cream-color, the PM; in blue, the ribosomes; and in pink, the discs.
(ZIP)

Movie S4 **A dual-axis tomogram from a 250 nm Lowicryl HM23 section.** The tilt series were acquired at 200 kV and the tomogram was filtered with Tomobflow. Flat clustered discs are visible perpendicularly distributed to the PM in a fragment of a cell.
(ZIP)
