# Supplementary figures and images for: The Stack: A New Bacterial Structure Analyzed in the Antarctic Bacterium Pseudomonas deceptionensis M1T by Transmission Electron Microscopy and Tomography
Source: PLoS One. 2013 Sep 9;8(9):e73297. doi: 10.1371/journal.pone.0073297 (PMC3767748; doi:10.1371/journal.pone.0073297)

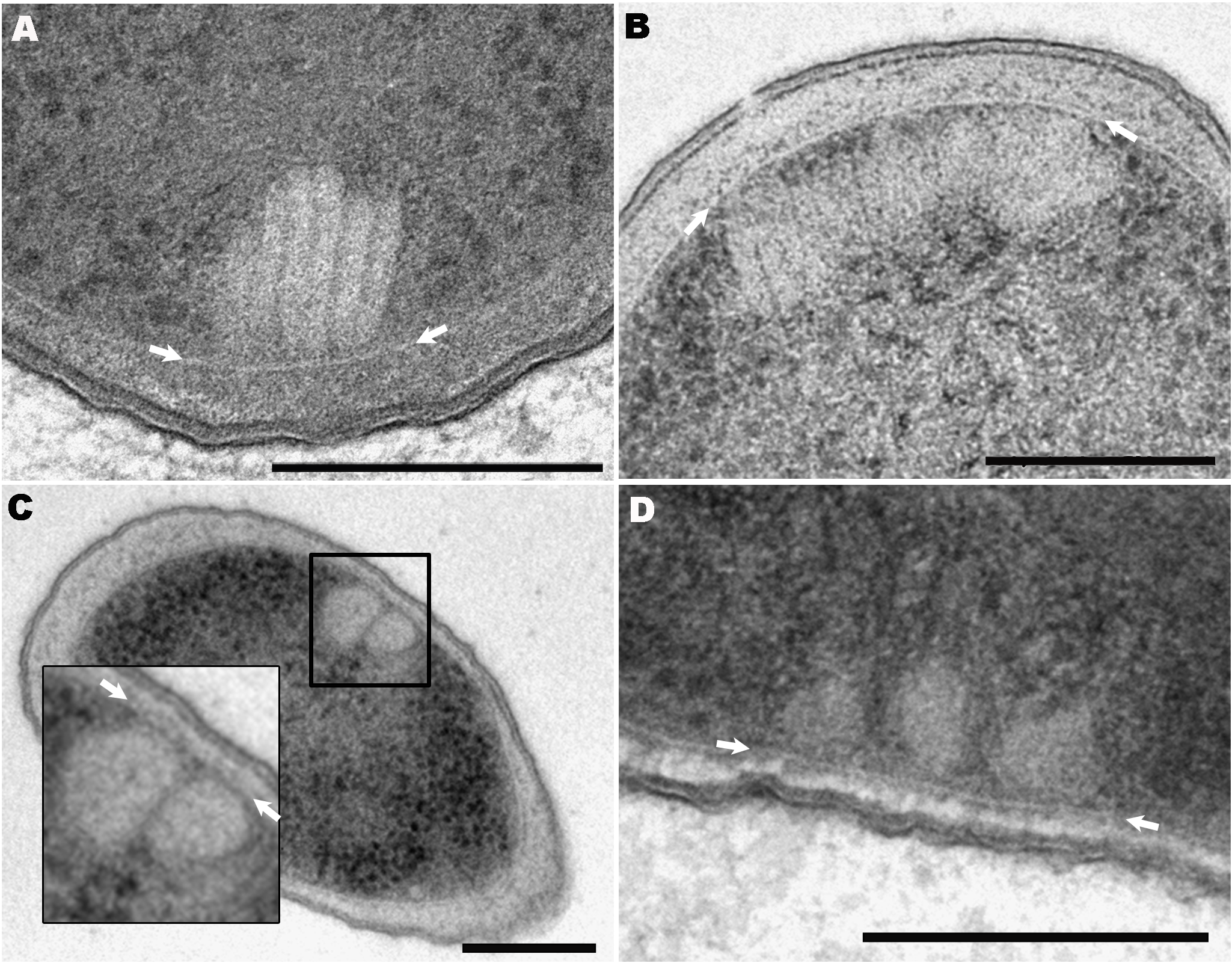

Supplement: Figure S1 — TEM visualization of stacks from P . deceptionensis M1T cells processed by HPF-FS. (A–D) 60 nm Epon sections. The PM is observed straight and uninterrupted and no continuity with stacks is observed in any case. Scale bars = 250 nm. (TIF) [file pone.0073297.s001.tif]
